# Leveraging Technology to Disentangle the Relationship Between Sleep and Cognition in Daily Life

**DOI:** 10.1093/geroni/igaf122.309

**Published:** 2025-12-31

**Authors:** Christopher Kaufmann, Todd Manini

**Affiliations:** University of Florida, Gainesville, Florida, United States; University of Florida, Gainesville, Florida, United States

## Abstract

There is strong interest in how poor sleep impacts cognition and neurodegenerative diseases like Alzheimer’s disease. Research across basic science, clinical, and epidemiological studies highlight a strong relationship between the two; however, questions remain about causality due to concerns about reverse causation. Despite the rigor of prior studies, the bidirectional nature of this relationship necessitates a nuanced understanding on a day-to-day and night-to-night basis within community settings. Recent technological advances, such as wearables and smartphones, offer new avenues to explore these daily and weekly dynamics. While accelerometers have been used to measure sleep for nearly four decades, modern wearables can now provide valuable insights, including sleep staging and nighttime breathing rate, factors which may significantly impact daily cognition. Traditionally, cognitive assessments have been conducted in the lab using formal testing; however, the proliferation of smartphones has enabled researchers to evaluate cognition in everyday settings. These new technologies present exciting possibilities to clarify the connection between poor sleep and neurodegeneration. However, it is essential to consider the limitations of data capture for accurate interpretation of the findings. This presentation will introduce how new technologies can enhance measurement of sleep and cognition in real world contexts, addressing both their strengths and limitations. The goal is to equip audience members with insights on effectively utilizing these tools in their own research with older adults. Various applications of these technologies will be demonstrated through practical examples.

